# Yield stability dataset of new orange fleshed sweet potato (*Ipomoea batatas L. (lam*)) genotypes in West Java, Indonesia

**DOI:** 10.1016/j.dib.2020.106297

**Published:** 2020-09-09

**Authors:** Haris Maulana, Sitaresmi Dewayani, M. Amir Solihin, Mahfud Arifin, Suseno Amien, Agung Karuniawan

**Affiliations:** a Faculty of Agriculture, Universitas Padjadjaran, Bandung, Indonesia; b Office of Food Crops and Horticulture of West Java Province, Indonesia

**Keywords:** AMMI, GGE biplot, Stability, Orange-fleshed sweet potato

## Abstract

There are many local varieties of sweet potatoes which are cultivated and consumed in Indonesia. The food industry which uses sweet potato as the main raw material has been developed in West Java. Demand for orange-fleshed sweet potato is high, but the supply of demand has not been fulfilled. This is because the varieties that are widely cultivated do not meet consumer standards and preferences, so new superior genotypes are needed following demand. Currently, selection of stable and high-yielding genotypes and accordance with consumer and industry preferences is one of the focuses of sweet potato research. Orange-fleshed sweet potato multi locations testing in accordance with consumer and industry preferences, can be used as a basis for consideration in the development program. The purpose of this study were to identify genotype by environment interactions (GEIs) and t select superior genotypes and to estimate yield stability across three locations in West Java, Indonesia. Combined analysis of variance (ANOVA) was used to determine significant differences between each genotype tested in term of yield and to estimated genotype by environment interactions (GEIs). Additive Main Effects and Multiplicative Interaction (AMMI), Genotype Plus Genotype by Environment Interactions (GGE) biplots, and Parametric and non-parametric stability measurements were used to determine yield stability from genotypes tested in all locations (Sumedang Regency, Bandung Regency, Karawang Regency). Data in this article showed that the genotypes, environments, and GEIs had an effect on sweet potato yields, with influences of 35.03%, 18.87%, and 46.01%, respectively. The results in this data also indicate that some new sweet potato genotypes have stable and high yields in three environments in West Java, Indonesia. So they were can be used for development in sweet potato breeding programs.

## Specifications Table

**Table:** 

Subject	Data Article (Agricultural and Biological Science)
Specific subject area	Agricultural and Biological Science (general), Agronomy and Crop Science
Type of data	Table Figure
How data were acquired	Data was obtained by conducted field observations in three different locations. Data tables and figures were obtained by analyzing raw data using Genstat 12th software, Microsoft Excel 2010, and STABILITYSOFT.
Data format	Analyzed
Parameters for data collection	The conditions were considered for data collection was environmental conditions of the experiment.
Description of data collection	This data was collected by measured the yield of new sweet potatoes planted at three different locations. Harvest was done when the plants are 18 weeks after planting. The yields of each genotype are weighed whole by used a digital scale. The observed trait was tuber yield per plot. Yields were converted in tons / ha
Data source location	City/Town/Region: Sumedang, Bandung, Karawang Country: Indonesia Latitude and longitude for collected samples/data: latitude 6 ° 55′00.6 "S, longitude 107 ° 46′18.3" E (Sumedang); latitude 7° 03′35.3 "S, longitude 107 ° 38′46.5" E (Bandung); latitude 6 ° 20′15.1 "S, longitude 107 ° 18′20.2" E (Karawang) Altitude: 753 m.a.s.l. (Sumedang); 996 m.a.s.l. (Bandung); 24 m.a.s.l. (Karawang)
Data accessibility	With the article

## Value of the Data

• This data set provides additional information about the effect of different environmental conditions on the yield of new orange-fleshed sweet potatoes.
• The dataset in this article provides information to researchers, farmers, and industry users, about the stability of new orange-fleshed sweet potato yields planted in West Java, Indonesia.
• The data provided can be useful in genetic studies and plant breeding programs, especially the stability of sweet potato yields, as well as for industrial users for the development of planting areas.

## 1 Data Description

Sweet potatoes usually have different yield potential if planted in diverse environments. Potential yield is one of the important characters in crops [1]. The yield can be also provided economic value to farmers, the community, and also industry users. In the food industry, information about suitable planting locations is one of the important things in the development of planting raw materials. Yields also play an important role in the development of sweet potatoes. This can also be a benchmark for farmers and industry in using certain varieties. Information about GEIs is very important in sweet potato breeding programs [2]. The existence of GEIs makes it difficult in the process of plants selection [2,3]. Stable and high yield are ideal genotypes expected by plant breeders and farmers [4,5]. This data set consists of information about the yields of 23 genotypes of new orange-fleshed sweet potatoes crossing which were planted in three different locations in West Java, Indonesia. The data presented in this article consists of three (3) figures and four (4) tables.

Table 1 shows the results of a combined variance analysis of genotypes tested in three environments. Table 2 presents the variation of yield from 23 sweet potato genotypes in three locations, while Table 3 shows the yield stability values in three environments with ASV and GSI models. Table 4 presented the parametric and non-parametric stability measurements for sweet potato genotypes. Based on the parametric stability in Table 4, ten genotypes in the current study showed higher *bi* values, indicating better adaptability of these genotypes to high-yielding environments. It is shown that MZ276, with *b* = 1.04, and MZ121 with *b* = 0.78, have slopes nearest to 1.00 among the 23 genotypes in the data set. On this criteria, MZ276 and MZ121 would be selected as the most stable of the 23 genotypes over the three environments in this multilocations test. Furthermore, these genotypes have the smallest deviation from regression on site index. This is measured by the deviation mean square of S^2^di of all genotypes and yield. In contrast, MZ202, MZ214, and MZ237 performs to be adapted to better environments. This is confirmed by the slope for this genotypes, *bi* = 2.12; *bi* = 3.48; and *bi* = 4.01, respectively, which is greater than 1 and also showed the highest yield out of 23 genotypes over three environments. Measurements S^(1)^, S^(2)^, S^(3)^, and S^(6)^ estimate MZ119, MZ127, and MZ270 as the most stable. NP^(1)^ estimated MZ270, MZ276, and Beta-2 as the most stable genotypes, while NP^(2)^, NP^(3)^, and NP^(4)^ selected mz119 as the most stable genotypes. Based on the combination of parametric and non-parametric stability measurements, genotypes that have a low average rank (AR) are stable genotypes [4].

**Table 1:** Combined variance analysis of 23 sweet potato genotypes.

Source	Df	SS	MS	F	F_prob	
Block(Environments)	6	0.36	0.06	0.30	0.97324	
Genotypes (G)	22	134.65	6.12	32.27	0.00000	**
Locations (E)	2	72.52	36.26	641.61	0.00000	**
Interactions (GEIs)	44	176.88	4.02	21.17	0.00000	**
Min. (ton/ha)	0.05					
Max. (ton/ha)	39.72					
Average (ton/ha)	11.93					
CV (%)	13.82					

Df= Degree freedom; SS= Sum of Square; MS= Mean of Square; Min. = Minimum Value; Max.= Maximum Value; ** *p*<0.01.

**Table 2:** Yield potential different on orange-fleshed sweet potato in each location.

Genotypes	Sumedang		Bandung		Karawang	
MZ119	17.40	cd	17.10	a	15.30	abc
MZ121	17.70	cd	18.70	a	7.65	def
MZ127	12.70	de	15.80	a	9.20	def
MZ128	11.20	de	17.10	a	6.40	def
MZ154	27.90	ab	2.90	de	5.75	efg
MZ202	33.50	ab	12.90	a	16.80	ab
MZ214	34.10	ab	15.00	a	0.10	i
MZ235	11.50	de	1.00	e	0.75	hi
MZ236	2.70	g	3.60	cd	2.65	gh
MZ237	37.23	a	4.50	bcd	2.85	gh
MZ247	23.50	bc	7.10	b	11.35	bcd
MZ270	6.10	f	1.10	e	0.10	i
MZ276	12.60	de	6.70	bc	2.55	gh
MZ290	13.70	de	2.00	de	0.10	i
MZ332	24.93	bc	2.70	de	19.20	a
MZ462	4.10	fg	4.00	bcd	17.80	a
MZ496	37.20	a	1.90	de	5.35	fg
Kidal	10.96	de	16.97	a	10.62	bcd
Rancing	13.70	de	17.71	a	16.34	ab
Beniazuma	10.35	e	19.00	a	9.83	cdef
Beta-2	11.36	de	13.52	a	9.20	cdef
Keriting Maja	12.22	de	18.18	a	8.68	def
AC-Putih	10.89	e	15.62	a	10.44	bcde
Kidal	17.40	cd	17.10	a	15.30	abc
Mean	17.28		10.22		8.22	
CV (%)	9.29		13.65		18.28	

Means followed by the same letter are not significantly difference, while those followed by different letters had significant difference at the 5% level by Duncan test; CV = coefficient of variation.

**Table 3:** Mean performance of sweet potato genotypes based on AMMI Stability Value (ASV) and Genotype Stability Index (GSI).

No.	Genotype	MY	RMY	IPCA[1]	IPCA[2]	ASV	RASV	GSI	RGSI
1	MZ119	16.60	2	0.81	−0.17	1.58	5	7	1
2	MZ121	14.68	8	0.54	1.22	1.60	6	14	2
3	MZ127	12.57	13	0.99	0.49	1.98	10	23	7
4	MZ128	11.57	16	1.13	1.06	2.44	12	28	18
5	MZ154	12.18	15	−1.90	−0.37	3.70	19	34	22
6	MZ202	21.07	1	−1.33	−0.62	2.65	16	17	4
7	MZ214	16.40	3	−2.02	2.22	4.50	21	24	9
8	MZ235	4.42	21	−0.33	−0.20	0.66	3	24	10
9	MZ236	2.98	22	0.99	−0.33	1.95	9	31	21
10	MZ237	14.86	6	−3.02	0.48	5.88	22	28	19
11	MZ247	13.98	9	−0.82	−0.76	1.76	7	16	3
12	MZ270	2.43	23	0.29	−0.20	0.60	2	25	13
13	MZ276	7.28	19	0.04	0.30	0.31	1	20	6
14	MZ290	5.27	20	−0.54	0.08	1.06	4	24	11
15	MZ332	15.61	5	−0.94	−2.52	3.11	18	23	8
16	MZ462	8.63	18	1.56	−2.56	3.96	20	38	23
17	MZ496	14.82	7	−3.09	−0.25	5.99	23	30	20
18	Kidal	12.85	12	1.35	0.39	2.64	15	27	16
19	Rancing	15.92	4	1.34	−0.33	2.62	14	18	5
20	Beniazuma	13.06	10	1.53	0.77	3.06	17	27	17
21	Beta-2	11.36	17	0.99	0.16	1.91	8	25	14
22	Keriting Maja	13.03	11	1.20	0.88	2.48	13	24	12
23	AC-Putih	12.32	14	1.25	0.24	2.43	11	25	15

MY= Mean yield; RMY=Rank of Mean Yield; IPCA = Interaction Principal Component AxisASV= AMMI Stability Value; RASV= Rank of ASV; GSI= Genotype Stability Index; RGSI= Rank of GSI.

**Table 4:** Yield stability based on parametic and non-parametric measures.

Genotype	Y	S⁽¹⁾	S⁽²⁾	S⁽³⁾	S⁽⁶⁾	NP⁽¹⁾	NP⁽²⁾	NP⁽³⁾	NP⁽⁴⁾	Wᵢ²	σ²ᵢ	s²dᵢ	bᵢ	
SP1	16.60	2.67	4.33	0.50	0.27	4.67	0.09	0.20	0.15	31.44	14.22	0.15	0.18	
SP2	14.68	7.33	30.33	3.71	0.69	7.33	0.21	0.38	0.45	49.27	23.98	6.73	0.78	
SP3	12.57	2.67	4.33	0.63	0.34	4.00	0.21	0.22	0.20	51.80	25.37	2.93	0.17	
SP4	11.57	7.33	32.33	5.54	1.09	10.33	0.57	0.63	0.63	87.28	44.80	8.02	0.17	
SP5	12.18	8.67	46.33	8.18	1.35	10.33	0.46	0.65	0.76	172.16	91.28	5.18	2.73	
SP6	21.07	6.00	24.33	2.75	0.64	7.67	0.16	0.33	0.34	92.13	47.45	4.97	2.12	
SP7	16.40	13.33	103.00	17.17	1.83	10.33	0.43	0.68	1.11	310.96	167.29	4.71	3.48	
SP8	4.42	5.33	16.33	7.00	1.86	3.67	1.15	0.61	1.14	5.91	0.24	0.39	1.26	
SP9	2.98	4.00	10.33	4.43	1.57	6.00	2.19	1.05	0.86	48.60	23.62	0.08	-0.03	
SP10	14.86	10.67	76.00	11.69	1.54	13.00	0.42	0.71	0.82	433.24	234.25	3.05	4.01	
SP11	13.98	4.67	14.33	1.87	0.57	5.33	0.15	0.33	0.30	45.27	21.79	4.15	1.60	
SP12	2.43	1.33	1.00	1.00	1.00	2.00	4.33	0.82	0.67	4.89	-0.32	0.01	0.67	
SP13	7.28	4.00	10.33	2.38	0.85	2.67	0.48	0.29	0.46	2.31	-1.73	0.32	1.04	
SP14	5.27	8.00	39.00	13.00	2.33	5.33	0.77	0.64	1.33	14.02	4.68	0.11	1.54	
SP15	15.61	12.00	86.33	11.26	1.35	8.33	0.32	0.55	0.78	194.53	103.53	27.24	1.29	
SP16	8.63	13.33	105.33	19.75	2.13	12.67	0.56	0.87	1.25	272.19	146.06	10.01	-1.11	
SP17	14.82	12.67	97.00	17.64	2.00	14.00	0.55	0.90	1.15	448.73	242.73	9.49	3.90	
C1	12.85	7.33	40.33	6.05	1.10	6.67	0.39	0.51	0.55	88.64	45.55	3.40	-0.20	
C2	15.92	4.67	16.33	1.85	0.53	8.00	0.17	0.42	0.26	86.68	44.47	0.33	-0.36	
C3	13.06	12.67	91.00	13.00	1.43	10.33	0.39	0.61	0.90	123.76	64.78	7.07	-0.28	
C4	11.36	3.33	8.33	1.47	0.59	3.00	0.44	0.31	0.29	46.04	22.21	1.27	0.10	
C5	13.03	7.33	34.33	4.79	0.93	9.00	0.32	0.45	0.51	85.44	43.79	6.56	0.07	
C6	12.32	7.33	37.00	6.17	1.17	4.33	0.38	0.51	0.61	74.47	37.78	2.23	-0.14	
Genotype	Y	S⁽¹⁾	S⁽²⁾	S⁽³⁾	S⁽⁶⁾	NP⁽¹⁾	NP⁽²⁾	NP⁽³⁾	NP⁽⁴⁾	Wᵢ²	σ²ᵢ	s²dᵢ	bi	AR
SP1	2	2	3	1	1	7	1	1	1	5	5	4	11	3.38
SP2	8	11	11	9	7	12	6	7	7	9	9	18	2	8.92
SP3	13	2	2	2	2	5	5	2	2	10	10	10	13	6.00
SP4	16	11	12	12	11	17	19	15	12	14	14	20	12	14.23
SP5	15	17	17	16	15	17	15	17	14	18	18	16	19	16.46
SP6	1	10	10	8	6	13	3	6	6	16	16	15	18	9.85
SP7	3	22	22	21	19	17	13	18	19	21	21	14	21	17.77
SP8	21	9	8	15	20	4	21	14	20	3	3	7	3	11.38
SP9	22	5	6	10	18	10	22	23	17	8	8	2	17	12.92
SP10	6	18	18	18	17	22	12	19	16	22	22	11	23	17.23
SP11	9	7	7	6	4	8	2	5	5	6	6	13	7	6.54
SP12	23	1	1	3	10	1	23	20	13	2	2	1	5	8.08
SP13	19	5	5	7	8	2	16	3	8	1	1	5	1	6.23
SP14	20	16	15	19	23	8	20	16	23	4	4	3	6	13.62
SP15	5	19	19	17	14	15	8	12	15	19	19	23	4	14.54
SP16	18	22	23	23	22	21	18	21	22	20	20	22	20	20.92
SP17	7	20	21	22	21	23	17	22	21	23	23	21	22	20.23
C1	12	11	16	13	12	11	11	10	10	15	15	12	10	12.15
C2	4	7	9	5	3	14	4	8	3	13	13	6	8	7.46
C3	10	20	20	19	16	17	10	13	18	17	17	19	9	15.77
C4	17	4	4	4	5	3	14	4	4	7	7	8	15	7.38
C5	11	11	13	11	9	16	7	9	9	12	12	17	16	11.77
C6	14	11	14	14	13	6	9	11	11	11	11	9	14	11.38

*Y* is Yield; S^(1)^,S^(2)^,S^(3)^,S^(6)^ is Nassar and Huehn (1987) [12] and Huehn (1990)[13]; NP^(1)^,NP^(2)^,NP^(3)^,NP^(4)^ is Thennarassu (1995) [14]; *Wᵢ*² is Wricke ecovalence [10]; *σ*²*ᵢ* is Shukla's stability variance [11]; *s*²*dᵢ, bi* is Eberhart and Russell (1966) [9]; AR is average rank's.

**Fig. 1:**
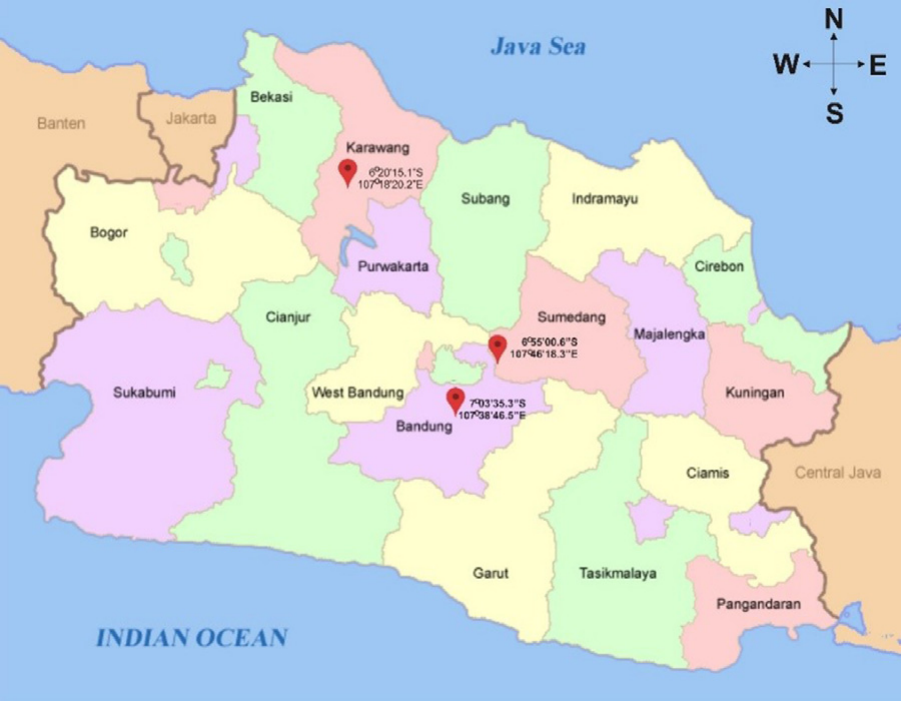
Field Trial Location in West Java, Indonesia.

**Fig. 2:**
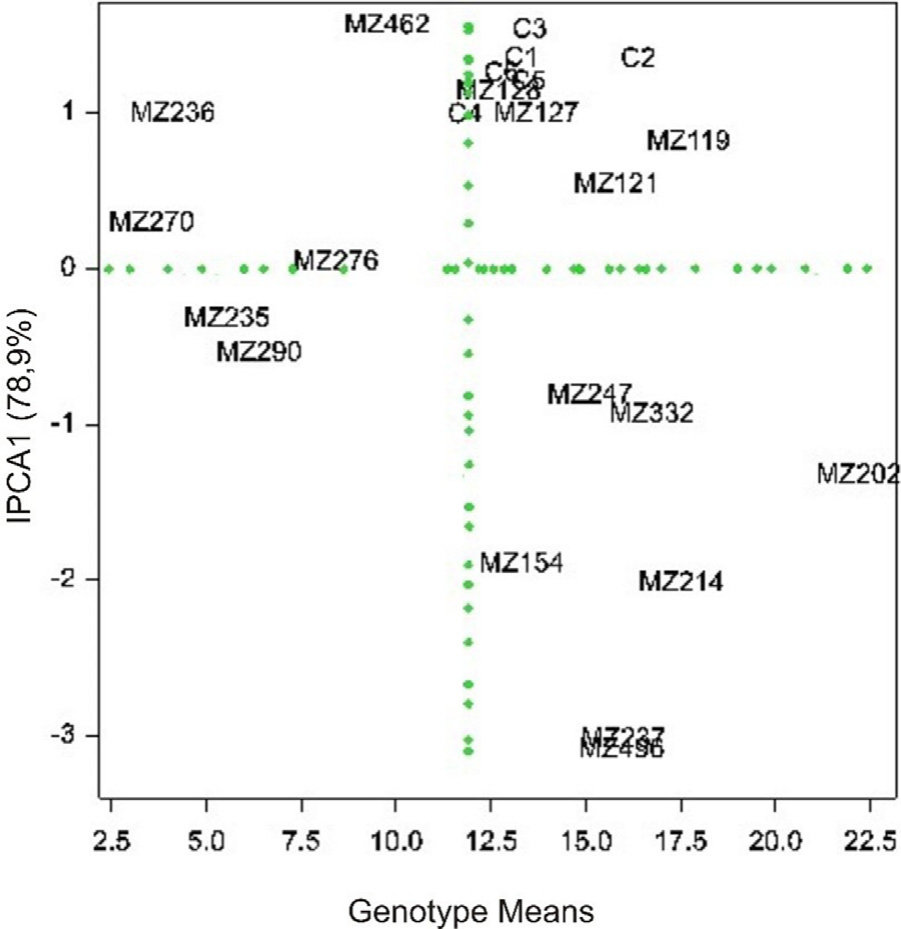
Biplot analysis of genotype by environment interaction based on AMMI1 model for the IPCA1 scores and sweet potato genotype in three location (1 = Sumedang regency, 2 = Bandung regency, and 3 = Kawarang regency) for yield character.

Fig. 1 shows the different planting locations in West Java, Indonesia. Fig. 2 shows the results of stability analysis used AMMI. In the Fig. 2, the horizontal line shows zero interaction with environment (PCA1). Sweet potato genotypes close to the line have a small GEIs effect or stable. The vertical center line represents the average value of sweet potato yield. Genotypes on the right-hand side have higher yields (above the overall average) compared to those on the left-hand side. Genotypes that approach the zero IPCA1 line are stated to be the most stable and high yield, they were MZ119, MZ121, MZ247, and MZ332.

**Fig. 3:**
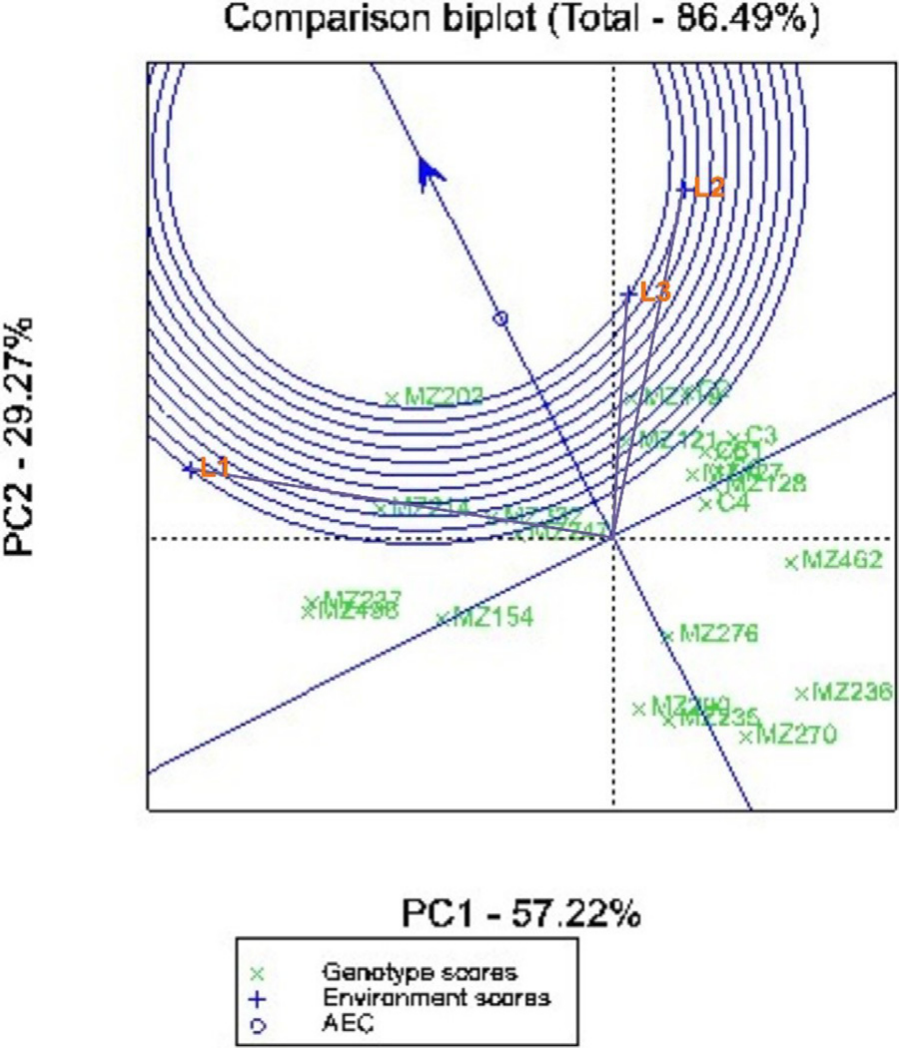
Rank's of the experimental location (L1 = Sumedang regency, L2 = Bandung regency, and L3 = Kawarang regency) based on discriminative and representativeness for sweet potato genotypes yield.

Fig. 3, showed the experimental location differ in discriminating ability and representativeness on the performance of sweet potato genotypes. The length of the experimental location vector from the biplot origin shows the discriminating ability of the location on superior genotypes for yield. The representativeness of the experimental location was indicated by the small angle between the experimental location and the average environmental axis. L3 (Karawang), has small angle to the average environmental compared (AEC), which means it was more representative than other locations. L1 (Sumedang), has the longest vector from the biplot origin, so it has good discriminating ability compared to other locations. L2 (bandung) has the second longest vector after L1 and has the second smaller angle to the AEC after L3. L2 and L3 both fall into the firts concentric circle of the ideal environment and closer to average environment compared L1. Thus, L2 had better discriminating ability and representativeness, and is an ideal location for evaluating the yield of sweet potato genotypes.

## 2 Experimental Design, Materials and Methods

### 2.1 Plant materials

The genetic materials used include seventeen new breeding genotypes, namely, MZ119, MZ121, MZ127, MZ128, MZ154, MZ202, MZ214, MZ235, MZ236, MZ237, MZ247, MZ270, MZ276, MZ290, MZ332, MZ462, MZ496, and six commercial varieties as checks, namely, Kidal (C1), Rancing (C2), Beniazuma (C3), Beta-2 (C4), Keriting Maja (C5), and AC Putih (C6). These tubers were previously selected based on consumer preferences.

### 2.2 Description of experimental locations

Field experiments of this study were conducted in Sumedang regency, Bandung regency, and Karawang regency (Fig. 1). Sumedang regency was located at latitude 6 ° 55′00.6 "S, longitude 107 ° 46′18.3" E and altitude 753 m.a.s.l. Bandung regency was located at latitude 7 ° 03′35.3 "S, longitude 107 ° 38′46.5" E and altitude 996 m.a.s.l. Karawang regency was located at latitude 6 ° 20′15.1 "S, longitude 107 ° 18′20.2" E and altitude 24 m.a.s.l.

### 2.3 Experimental design and planting

Field experiments were carried out in three locations, Sumedang regency, Bandung regency, and Karawang regency (Fig. 1). Seventeen (17) new sweet potato genotypes and six (6) check varieties at each trial location were planted using a Randomized Block Design that was repeated 3 times. Each genotype was planted in blocks measured 25 × 100 centimeters in a row along 5 m. The first planting was carried out in Sumedang regency on January–May 2017. This was followed by planting in Bandung regency on January–May 2018. In Karawang regency was carried out on February–July 2018.

### 2.4 Data collection

This data was collected by measured the yield of new sweet potatoes planted at three different locations. Harvest was done when the plants are 18 weeks after planting. The yields of each genotype are weighed whole by used a digital scale.The observed trait was tuber yield per plot. The data were collected at the time of harvest. The weight (kg) of sample obtained from a 5 m^2^ plot of each genotype. Yields were converted in tons/ha.

### 2.5 Data analysis

An estimation of the GEIs was carried out for all genotypes. The statistical model for combined ANOVA of the environments was as follows:

(1) $$Y_{ijkl}=\mu +G_{i}+E_{j}+GE_{ij}+R_{k(j)}+B_{l(k)}+\varepsilon_{ijkl}$$

where *Y_ijkl_* is the value in plot *l* of genotype *i*, and the value in location *j* of each replication *k; μ* is the grand mean; *G_i_* is the influence of genotype *i; E_j_* is the influence of the location; *GE_ij_* is the influence of interaction between genotype *i* and location *j; R_k(j)_* is the influence of replicate *k* on location *j; B_l(k)_* is the influence of repeat k on plot *l*; and *ε_ijkl_* is the influence error of genotype *i* in plot *l* and repeat *k* of location *j*, respectively.

Genotype by environment interactions (GEIs) were estimated with combined analysis of variance (ANOVA) using the GenStat 12th statistical software, so as to determine significant differences between each genotype tested in the three environments. The yield difference of each genotype by Duncan test method at a probability level of 5%.

AMMI model were analyzed with GenStat 12th statistical software. This analysis used to determine the GEIs, assess the adaptability and stability of genotypes planted in three locations. Identification of stable genotypes using AMMI following the study of [6]:

(2) $$Y_{ijk}=\mu +Gi+E_{j}+\sum_{k=1}^{m}\left(\lambda_{k\;}\alpha_{\text{ik}}\gamma_{\text{jk}}\right)+\rho_{\text{ijr}}$$

where: *Y_ijk_* is the yield in location *j*  from genotype i of replication *k, μ* is the average of grand yield, *G_i_*  is the influence of genotype *i*, *E_j_* is the influence of the location *j*, *λ_k_* is the value of primer component *k*, *α_ik_* and *γ_jk_*  were the vector score for the genotype *i* and location *j* to component *k*, *ρ_ijr_*  is a mistake from genotype *i* and location *j*

While ASV was estimated following the study of [7]:

(3) $$\text{ASV}=\sqrt{\frac{ss\;IPCA1}{ss\;IPCA\;2}{\left(IPCA\;1\right)}^{2}+{\left(IPCA\;2\right)}^{2}}$$

Were: ss IPCA1, ss IPCA2 were the sum of square in IPCA 1 and 2, which shows the score of the main component because of the high contribution in genotype by location interactions. IPCA1 and IPCA2 were the first and second from IPCA scores for each genotype from the AMMI analysis.

The value of the Genotype Stability Index (GSIg) of each sweet potato genotype was calculated based on the *g*th genotype rank in three environments based on ASV Rank (RASVg) and *g*th genotype rank based on the average yield in three environments (RMYg) with the following equation:

(4) GSIg=RASVg+RMYg

GSIg was analyzed using Microsoft excel 2010.

The model for a GGE biplot following [8] with the formula:

(5) $$\;{\bar{Y}}_{\text{ij}}-\;\mu_{i}-\;\beta_{j}=\;\sum_{k=1}^{t}\lambda_{k}\alpha_{ik}\gamma_{jk}+\varepsilon_{ij}$$

where *Ῡ_ij_* is the yield performance in location *j* from genotype *i, μ_i_* is the overall average yield, *β_j_* is the influence of location *j, k* is the number of primer components; *λ_k_* is the singular value from the primer component *k*; and *α_ik_* and *γ_jk_* is the value of genotype *i* and location *j* for primer component *k; ε_ij_* is the error of genotype *i* in location *j*. GGE Biplot was analyzed using the GenStat 12th statistical software. This analysis was used to determine the ability of discriminating and representativeness of the field trials on sweet potato genotypes.

Identification of stable genotypes among stable was conducted using parametric and non-parametric stability models. The Eberhart and Russell method [9]uses to identify stability genotype based on linear regressions. If the variance deviation (*S*^2^d*i*) = 0, and the regression slope (bi) = 1 indicated the genotype was stable. Wricke's Ecovalence (*W_i_*^2^) following [10] with the formula:

(6) $$W_{i}^{2}=\sum {\left(X_{ij}-{\bar{X}}_{i.}-{\bar{X}}_{.j}+{\bar{X}}_{..}\right)}^{2}$$

Shukla's stability variance (*σ*^2^*i*) following [11]with the formula:

(7) $$\sigma_{i}^{2}=\left|\frac{p}{\left(p-2\right)\left(q-1\right)}\right|W_{i}^{2}-\;\frac{\sum W_{i}^{2}}{\left(p-1\right)\left(p-2\right)\left(q-1\right)}$$

Where *x*_ij_: the total yield of genotype *i* in location *j*; ${\bar{X}}_{i.}$: the average yield of genotype *i*; ${\bar{X}}_{.j}$: Average yield of the location *j*; ${\bar{X}}_{..}$: the grand mean; *p* and *q*: the numbers of genotypes and location.

Stability non-parameters (*S*^(^*^i^*^)^) models following [12,13] with the formula:

(8) $$S_{i}^{\left(1\right)}=2\sum_{j}^{n-1}\frac{\sum_{j^{\prime }=j+1}^{n}\left|r_{ij}-r_{ij}^{\prime }\right|}{\left[N\left(n-1\right)\right]},$$

(9) $$S_{i}^{\left(2\right)}=\;\frac{\sum_{j=1}^{n}{\left(r_{ij}-{\bar{r}}_{i.}\right)}^{2}}{\left(N-1\right)},$$

(10) $$S_{i}^{\left(3\right)}=\;\frac{\sum_{j=1}^{n}{\left(r_{ij}-{\bar{r}}_{i.}\right)}^{2}}{{\bar{r}}_{i}},$$

(11) $$S_{i}^{\left(6\right)}=\;\frac{\sum_{j=1}^{n}\left|r_{ij}-{\bar{r}}_{i.}\right|}{{\bar{r}}_{i.}}$$

where *r_ij_*: rank of stability from genotype i in the location *j*; ${\bar{r}}_{i.}$: mean rank across all location for each genotype; and *N*: number of location. Stability parameters (NP^(i)^) following [14] with the formula:

(12) $$NP^{\left(1\right)}=\frac{\sum_{j=1}^{n}\left|r_{ij}^{*}-M_{di}^{*}\right|}{N},$$

(13) $$NP^{\left(2\right)}=\frac{\left[\sum_{j=1}^{n}\left|r_{ij}^{*}-M_{di}^{*}\right|/M_{di}\right]}{N},$$

(14) $$NP^{\left(3\right)}=\frac{\sqrt{\frac{\sum {\left(r_{ij}^{*}-r_{i.}^{*}\right)}^{2}}{N}}}{{\bar{r}}_{i.}},$$

(15) $$NP^{\left(6\right)}=\frac{2x\left[\sum_{j=1}^{n-1}\sum_{j^{\prime }=j+1}^{n}\left|r_{ij}^{*}-r_{i.}^{*}\right|/{\bar{r}}_{i.}\right]}{N\left(N-1\right)}$$

where $r_{ij}^{*}$: stability rank in location *j* from genotype i based on adjusted data; $M_{di}^{*}$: median rank for adjusted data; *M_di_:* Original data from the same parameters. *N*: number of location. To calculate stability genotypes based on parametric and non-parametric statistic models, we used online software STABILITYSOFT [15].

## Declaration of Competing Interest

The authors declare that they have no known competing financial interests or personal relationships which have, or could be perceived to have, influenced the work reported in this article.

## Appendix Supplementary materials

Image, application 1

Image, application 2
